# Fatal Paraneoplastic Embolisms in Both Circulations in a Patient with Poorly Differentiated Neuroendocrine Tumour

**DOI:** 10.1155/2013/739427

**Published:** 2013-12-30

**Authors:** A. Busch, S. Tschernitz, A. Thurner, R. Kellersmann, U. Lorenz

**Affiliations:** ^1^Department of General, Visceral, Vascular and Paediatric Surgery, Julius Maximilians University of Wuerzburg, Oberduerrbacher Street 6, 97080 Wuerzburg, Germany; ^2^Institute of Pathology, Julius Maximilians University of Wuerzburg, Oberduerrbacher Street 6, 97080 Wuerzburg, Germany; ^3^Institute of Radiology, Julius Maximilians University of Wuerzburg, Oberduerrbacher Street 6, 97080 Wuerzburg, Germany

## Abstract

Arterial embolism with lower limb ischemia is a rare manifestation of paraneoplastic hypercoagulability in cancer patients. We report a unique case of fatal thromboembolism involving both circulations associated with a poorly differentiated neuroendocrine tumor of the lung with rapid progress despite high doses of unfractioned heparin and review the current literature on anticoagulative regimen in tumour patients.

## 1. Introduction

Limb ischemia due to arterial embolism in cancer patients has been reported for phaeochromocytoma, malignant melanoma, angiosarcoma, or cardiac tumour patients [1–3]. Single cases have also been linked to pancreatic adenocarcinoma or carotid body paraganglioma [4, 5]. Tumour associated venous thrombosis with pulmonary embolism on the other hand is much more common. Compared to a normal population, cancer patients bear a thirty times greater risk of deep vein thrombosis [6].

Vascular tumour invasion, metastatic spread, and fragmentation of cardiac masses need to be distinguished from paraneoplastic effects like catecholamine associated vasospasm, protein precipitation, and hypercoagulability [7]. The latter is of largely unknown origin and is responsible for about 10% of cancer-related thromboembolisms [8, 9]. Additionally, catecholamine independent arterial vasospasm leading to limb ischemia has been recently reported in a patient with well-differentiated pulmonary neuroendocrine carcinoma [10].

Embolism suggestive of Trousseau syndrome may occur as initial symptom resulting in tumour diagnosis, as complication alongside disease progression or may be triggered by specific chemotherapy agents [11, 12]. Thromboembolism is the second leading cause of death in cancer patients [13].

Here we report the unique case of a patient diagnosed with a multilocular metastasized neuroendocrine tumour (NET) of the lung suffering from progressive arterial embolisms in both circulations leading to bilateral lower limb ischemia, pulmonary embolism, stroke, and finally death five weeks after the initial bout of chemotherapy despite high doses of unfractioned heparin.

## 2. Case Report

A 58-year-old Caucasian male was admitted to the Oncology Department with polyuria and weight loss of seven kilos in the last two weeks. Risk factors were arterial hypertension and cigarette smoking. Syndrome of inappropriate antidiuretic hormone secretion (SIADH) with hyponatremia was noticed and tumour search detected metastatic liver disease and enlarged hilar lymph nodes. Blood analysis revealed elevated levels for neuron-specific enolase (NSE), carcinoembryonic antigen (CEA), cytokeratin-fragment (Cyfra), and liver biopsy specified metastases of a small cellular, poorly differentiated neuroendocrine tumour (NET). Further staging via FDG PET disclosed the primary tumour near the left lung hilus with positive mediastinal and hilar lymph nodes, diffuse osseous filiarisation, and a sole metastasis in the right adrenal gland. Oncology board consensus decision was made for palliative chemotherapy with cisplatin and etoposid (80 mg/sqm, 120 mg/sqm, resp.) as common for dedifferentiated NET.

**Figure 1:**
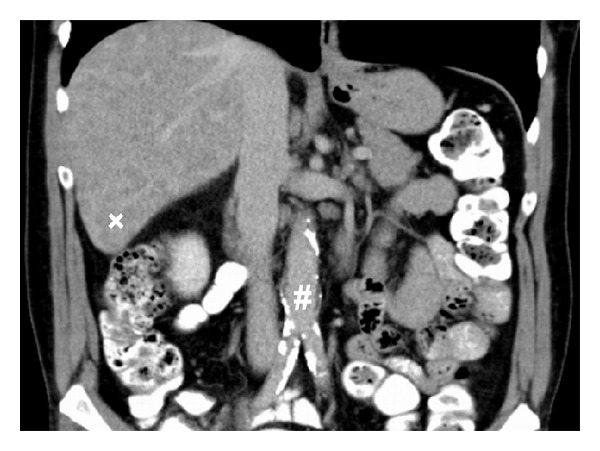
Abdominal CT-scan showing diffuse calcifications of the infrarenal aorta and aortic bifurcation (#) as well as mild ectasia (3 cm). Note the diffuse hepatic metastatic disease (x).

Two weeks after the initial bout of chemotherapy the patient presented in the Surgical Department with incomplete ischemia of the left lower limb. Immediate interventional recanalization failed and open embolectomy of the tibiofibular trunk with consecutive venous patch plasty was performed. Histological examination of the embolus showed no malignancy. Three days later reischemia of the same leg occurred and the operative procedure was repeated. Search for embolic origin by CT-scan disclosed clinically inapparent pulmonary embolism in three lobes. Mural thrombus of the infrarenal aorta with perifocal aortic wall calcifications appeared identical to the initial staging CT (Figure 1). All tumour manifestations showed downsizing in response to the previous systemic chemotherapy. High doses of unfractioned heparins (up to 40.000 IE/d) were administered for anticoagulation. Aspirin was given for platelet inhibition. Activated partial thromboplastin time presented sufficiently prolonged with normal levels of antithrombin III when symptoms began.

**Figure 2:**
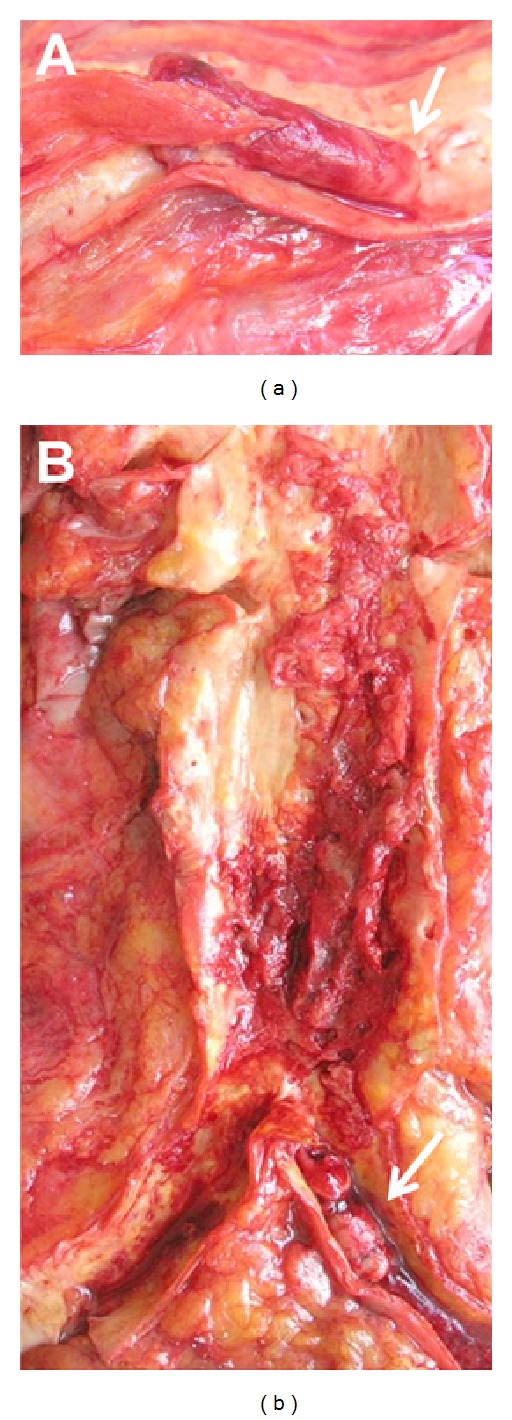
Pictures taken after autopsy of the right carotid bifurcation (a) with occluding thrombus in both branches. Please note the typical lines of Zahn at the arterial thrombus as a sign of continuing thrombus formation (arrow). The infrarenal aorta (b) shows multiple ulcerated arteriosclerotic plaques with mural thrombus luminal occlusion of the left common iliac artery (arrow).

Again four days later, transfemoral and pedal embolectomy with multilocular patch plasties of all native crural arteries as ultima ratio due to complete ischemia had to be performed. Decision was made for major amputation as symptoms recurred again two days later. After clinical signs of ischemia of the contralateral limb and respiratory impairment, all parties agreed upon the best supportive care concept. The patient died three days later after an additional stroke with left-sided hemiplegia. Autopsy revealed right-sided heart failure due to new severe pulmonary embolism as cause of death, as well as heavy thrombotic alterations of all major arterial vessels (Figure 2).

## 3. Discussion

To our best knowledge this is the first case with fulminant arterial embolisms involving the lung, brain, and both legs within a two-week period despite high doses of unfractioned heparins as paraneoplastic symptoms of a multilocular metastasized NET.

Initially, calcifications of the infrarenal aorta with intraluminal thrombus were suggested as origin of embolism and protective stenting was discussed but not conducted due to the rapid chain of events (Figures 1 and 2). Retrospectively with additional embolism upstream, the abdominal aorta as well as the lesser circulation would have been oblivious.

Conclusively, paraneoplastic or chemotherapy-triggered hypercoagulability remains as possible explanations for generalized thromboembolic disease.

Paraneoplastic syndromes occur in about 10% of malignant diseases and are defined as symptom complexes that cannot be readily explained by either the local or distant spread of cancer cells or specific secretory products [14]. Constitutive overexpression of procoagulative tissue factor and phosphatidylserine could be shown to be responsible for arterial and venous embolism in human malignant melanoma and phaeochromocytoma cell lines [12, 15]. Oncogene activation and tumour suppressor gene inactivation such as PTEN, MET, or p53 are thought to upregulate clotting pathways [16]. Activated endothelium in melanoma patients seems to additionally promote platelet activation [17]. Neural crest cell origin of NET might be a possible explanation for the involvement of many different pathways resulting in coagulation disorders [18].

Cisplatin, as part of the chemotherapeutic regimen among other substances such as thalidomide, is known to trigger thromboembolism in cancer patients with different nonrelated tumour entities [19–21]. The DNA-crosslinking drug is thought to provoke endothelial dysfunction and platelet activation by phosphatidylserine expression [22]. On the other hand, cisplatin based chemotherapy could be shown to improve symptoms of disseminated intravascular coagulation in a patient with metastasized gastric adenocarcinoma as treatment of the underlying malignancy [23].

Some authors have reported secondary heparin-induced thrombocytopenia (HIT) of type II in cancer patients suggesting a higher incidence due to bone marrow infiltration [24, 25]. HIT II antibody diagnostic was negative and HIT-related white-clot syndrome was not noticed at autopsy [26]. However, platelet count dropped to <50 × 10^9^/litre as the patient worsened rapidly.

In terms of which anticoagulant drug to administer in paraneoplastic embolism, low-molecular-weight heparin (LMWH) seems to be superior compared to unfractioned heparin (UFH) and vitamin K antagonists. This assumption is mainly supported by studies with patients suffering from deep vein thrombosis [27, 28]. Regarding arterial embolism no substantial clinical studies or guidelines have been published until now. Some authors suggest UFH or LMWH in combination with the direct thrombin inhibitor fondaparinux [6, 29]. In this context, UFH is known to affect more different sites in the coagulation cascade as LMWH [30]. The role of platelet inhibition is unclear.

## 4. Conclusion

Lower limb ischemia in tumour patients is a severe paraneoplastic complication and might be the first clinical sign in a fast chain of overwhelming thromboembolic events. Tumours of neural crest origin seem to bear a high risk of hypercoagulation. Adequate surgical intervention and sufficient anticoagulation are the only treatment options and may be still too late. Further substantial research on anticoagulative treatment in tumour patients suffering from severe thromboembolic complications is needed.
